# Identification of Wheat Glutamate Synthetase Gene Family and Expression Analysis under Nitrogen Stress

**DOI:** 10.3390/genes15070827

**Published:** 2024-06-22

**Authors:** Songshuo Li, Bo Jiao, Jiao Wang, Pu Zhao, Fushuang Dong, Fan Yang, Chunhong Ma, Peng Guo, Shuo Zhou

**Affiliations:** 1 School of Biological Science and Engineering, Hebei University of Science and Technology, Yuxiang Street 26, Shijiazhuang 050018, China; 15614113343@163.com; 2 Hebei Key Laboratory of Plant Genetic Engineering, Institute of Biotechnology and Food Science, Hebei Academy of Agriculture and Forestry Sciences, Shijiazhuang 050051, China; jiaobo1206@163.com (B.J.);

**Keywords:** glutamate synthase, wheat, N stress, tissue specificity

## Abstract

Nitrogen (N), as the main component of biological macromolecules, maintains the basic process of plant growth and development. GOGAT, as a key enzyme in the N assimilation process, catalyzes α-ketoglutaric acid and glutamine to form glutamate. In this study, six *GOGAT* genes in wheat (*Triticum aestivum* L.) were identified and classified into two subfamilies, *Fd-GOGAT* (*TaGOGAT2s*) and *NADH-GOGAT* (*TaGOGAT3s*), according to the type of electron donor. Subcellular localization prediction showed that TaGOGAT3-D was localized in mitochondria and that the other five TaGOGATs were localized in chloroplasts. Via the analysis of promoter elements, many binding sites related to growth and development, hormone regulation and plant stress resistance regulations were found on the *TaGOGAT* promoters. The tissue-specificity expression analysis showed that *TaGOGAT2s* were mainly expressed in wheat leaves and flag leaves, while *TaGOGAT3s* were highly expressed in roots and leaves. The expression level of *TaGOGATs* and the enzyme activity of TaGOGAT3s in the leaves and roots of wheat seedlings were influenced by the treatment of N deficiency. This study conducted a systematic analysis of wheat *GOGAT* genes, providing a theoretical basis not only for the functional analysis of *TaGOGATs*, but also for the study of wheat nitrogen use efficiency (NUE).

## 1. Introduction

Nitrogen (N) plays a key role in the anabolic process of proteins, nucleic acids, chlorophyll, hormones, and other substances that participate in the construction of living organisms [1]. Wheat, as one of the three major grain crops, currently covers about 200 million ha in the world, providing a lot of nutrition for people [2]. The use of N fertilizer is very important for increasing wheat yield, but it also places huge burdens on the environment such as soil acidification and water eutrophication [3]. It is estimated that around two-thirds of the N fertilizer used to grow cereals is wasted worldwide [4]. Improving the nitrogen use efficiency (NUE) of wheat can reduce the use of fertilizer, and reduce the production costs of farmers [5]. Therefore, it is of great significance to further study the mechanisms of N absorption, transport, and the metabolism of wheat, for alleviating human food needs and reducing environmental pollution.

Plant roots absorb inorganic N from the soil mainly in the form of nitrate (NO_3_^−^) and ammonium (NH_4_^+^). Ammonium is the main inorganic N source for plant growth and development [6]. Plants take up ammonium in soil through ammonium transporter (AMT)-mediated transport pathway [7]. Nitrate in soil is absorbed by plants through two transport systems, the high-affinity and low-affinity transport systems (HATS and LATS); the selection of a transport pathway depends on its concentration in the soil [8]. Nitrate in plants can be reduced to form nitrite, and then ammonium is produced by the catalytic reaction of nitrite reductase [9,10]. Ammonium in plants can participate in the GS–GOGAT cycle, and the generation of glutamine and glutamate can be used to join in the biosynthesis of other amino acid derivatives, and ultimately participate in the growth and metabolism of plants [11,12].

According to the classification of electron donor types, GOGAT can be classified into two categories: NADH-GOGAT and Fd-GOGAT, which use reduced ferredoxin (Fd) and nicotinamide adenine dinucleotide (NADH) as electron donors, respectively [13]. Fd-GOGAT is a monomeric protein with a molecular weight ranging from 145 kDa to 180 kDa [14] and is highly expressed in photosynthetic tissues. Its activity can be significantly induced by light or exogenous sucrose [15,16]. NADH-GOGAT is a protein with a molecular weight of about 200 kDa and can be detected in the non-photosynthetic tissue of plants [13,15].

Many studies have demonstrated the function of GOGAT in plants. In rice, the phenotypic characteristics of the *OsFd-GOGAT* mutant plants showed chlorosis under natural conditions, and slight premature aging under low light treatment [17]. Overexpression of *NADH-GOGAT* (EC *1.4.1.14*) in rice could increase the grain weight, and affect plant nitrogen-related utilization [18]. However, in corn, the *NADH-GOGAT* gene has the opposite effect. Overexpression of *NADH-GOGAT* gene influences shoot biomass accumulation. However, there are no significant effects on grain yield. Overexpression of *NADH-GOGAT* increases glutamate-derived amino acid contents and results in decreased concentrations of glusose-6-P, arabinose and fructose-6-P, which ultimately affect the generation of the C skeleton required for GOGAT synthesis. Finally, the primary carbon and N metabolism balance of maize is affected [19]. CRISPR/Cas9-mediated targeted mutagenesis of the *Fd-GOGAT* suppressor ARE1 (abnormal cytokinin response1 repressor1) [20], delayed senescence and increased wheat yield in the field [21].

*GOGAT* plays an important role in grain yield and quality by participating in key N metabolism processes. At present, many studies have been conducted on maize, rice, durum wheat, etc., but the related research on wheat is neither systematic nor in-depth. Therefore, in this study, bioinformatics was used to identify and analyze the members of the *GOGAT* gene family in bread wheat. The temporal and spatial expression patterns of *TaGOGATs* were analyzed by RT-qPCR in wheat seedlings under N deficiency treatment and different tissues of wheat under normal N concentrations at the filling stage. The enzyme activity in leaf and root tissues was analyzed under an N hunger treatment. Through the above analyses, a regulation model of wheat *GOGAT* under N stress was preliminarily studied.

## 2. Materials and Methods

### 2.1. Identification of TaGOGAT Gene Family Members

In this study, members of the wheat glutamate synthase gene family were identified by Hidden Markov Model (HMM) screening and BLAST comparison. Glutamate synthase gene family information files were downloaded from the Ensembl database (http://plants.ensembl.org/index.html (accessed on 12 January 2024)). The HMM file for the GOGAT conservative domain (PF01645) was obtained from the Pfam database (https://www.ebi.ac.uk/interpro (accessed on 13 March 2024)). The members of the *TaGOGAT* gene family were identified using HMMER 3.0 software. It has been reported that the *GOGAT* gene is highly conserved across species [22]. The gene sequences of three *GOGATs* (AT5G53460, AT5G04140, AT2G41220) from *Arabidopsis* and four *GOGATs* (KAI9392052, KAI9380114, KAI9384106, PNS99918) from *Populus* were downloaded from the NCBI (National Center for Biotechnology) database (https://www.ncbi.nlm.nih.gov/ (accessed on 13 March 2024)). The NCBI-BLAST method was used to search for the homologous proteins of GOGAT in wheat. The same proteins obtained by the two methods were taken as the preliminary identification results. Using the Batch CD—Web Search Tool (https://www.ncbi.nlm.nih.gov/Structure/bwrpsb/bwrpsb.cgi (accessed on 9 March 2024)) and SMART website (https://smart.embl.de/ (accessed on 9 March 2024)), six *TaGOGAT* gene family members were identified by removing the proteins that did not possess complete conserved domains. Information on the gene sequence, protein-coding sequence (CDS), amino acid sequence, gene position on chromosome, etc., was queried in the Ensembl database. The protein molecular weight, isoelectric point, hydrophobicity and instability coefficient were obtained through the ExPASy online analysis website (https://www.expasy.org/ (accessed on 14 March 2024)). The WoLF PSORT II website (https://www.genscript.com/ (accessed on 8 May 2024)) was used for subcellular localization prediction. The protein transmembrane structure was analyzed based on the TMHMM 2.0 website (https://www.healthtech.dtu.dk/ (accessed on 6 April 2024)). Chromosome location mapping was carried out by TBtools-II software v2.096.

### 2.2. Phylogenetic Tree Analysis of GOGAT and Ka/Ks Ratio Calculation

The amino acid sequences of TaGOGATs and its homologous genes in *Arabidopsis* and *Populus* were analyzed by MEGA 11. The optimal algorithm model was determined as WAG + G + I + F, the Bootstrap value was selected as 1000, and the maximum likelihood (ML) method was used for phylogenetic tree analysis. TBtools-II software plug-in (Simple Ka/Ks Calculator (NG)) was used to calculate the Ka/Ks ratio of the *TaGOGATs* gene, and Excel 2016 software was used to draw the scatter plot for the analysis of the selection pressure during the evolution process.

### 2.3. Conserved Motif Identification and Gene Structure Analysis

MEME 5.5.5 (https://meme-suite.org/meme/ (accessed on 27 March 2024)) was used to identify *GOGAT* conserved motifs in *Arabidopsis*, *Populus* and wheat (*T. aestivum*). The number of identified motifs was set to 10. Gene annotation files were downloaded from the Ensembl database. The Batch Web CD-Search Tool was used to obtain the information on the GOGAT protein conserved domain, and the gene structure map was drawn by TBtools-II software (Gene Structure View (Advanced)).

### 2.4. Collinearity Analysis of TaGOGATs Gene

The collinearity analysis plug-in (one Step MCScanX) of TBtools-II software was used to analyze the collinearity within species, based on the MCScanX algorithm kernel.

### 2.5. Prediction Analysis of Promoter Cis-Acting Elements

2 kb upstream sequence of *GOGAT* genes were downloaded from the Ensembl database, using the PlantCARE website (https://bioinformatics.psb.ugent.be/webtools/plantcare/html/ (accessed on 15 March 2024)) to predict the *cis*-acting elements of promoters. R language was used to draw heat maps to analyze the discrepancy in the type and number of *cis*-acting elements between different promoters.

### 2.6. TaGOGATs Amino Acid Sequence Alignment and Protein Tertiary Structure Analysis

The amino acid sequence alignment analysis of TaGOGATs was performed using the ESPript 3.0 website (https://espript.ibcp.fr/ESPript/cgi-bin/ESPript.cgi (accessed on 16 April 2024)). Using the SWISS-MODEL website (https://swissmodel.expasy.org/ (accessed on 5 May 2024)), the protein tertiary structure was predicted, and the optimal homologous protein model was found based on the homologous alignment method.

### 2.7. Plant Material and Gene Expression Analysis

The roots, stems, leaves, flag leaves and seeds of Chinese spring wheat at the filling stage were selected as samples for tissue-specific gene expression analysis. Wheat seeds with full grains and a relatively consistent size were selected and placed on the germination tray, kept in the dark for 2 days at 25 °C to permit germination, and then cultured under 16 h of light and 8 h of darkness for 4 days. After removing endosperm, the wheat seedlings were transferred to a 96-well plastic box (10.5 × 7 × 4 cm) and hydroponic with a modified Hoagland nutrient solution (Macro-nutrition: 1 mM KH_2_PO_4_, 4 mM CaCl_2_, 2 mM MgSO_4_·7 H_2_O, 5 mM KCl, 8 mM NH_4_NO_3_; Fe and Micro-nutrition was consistent with normal Hoagland solution, NH_4_NO_3_ as the only N source) for 3 days; ddH_2_O was used every day to ensure a constant volume of culture solution, and N deficiency culture was performed when they reached 2 leaves and 1 heart. The seedlings with similar growth were selected, and the root tissue was washed with ddH_2_O and dried with filter paper. Wheat seedlings were transferred to the modified Hoagland nutrient solution (exclude NH_4_NO_3_) for N starvation culture. Leaf and root tissues were collected after treatment for 0, 0.5, 1.0, 1.5, 2.0, 3.0, 6.0 and 24.0 h, respectively, as samples for *TaGOGATs* gene expression pattern analysis under N stress. All samples were frozen using liquid N after sampling, and stored at −80 °C. Each sample contained 4 biological replicates. Trizol reagent (Invitrogen, Waltham, MA, USA) was selected for total RNA extraction [23]. The NanoDrop 2000 (Thermo Scientific, Waltham, MA, USA) was used to determine the RNA concentration of the samples. A total of 1 μg RNA was taken from each sample for reverse transcription, and the reverse transcription reagent was HiScript RT SuperMix (Vazyme, Nanjing, China). The generated cDNA was used as the template for the RT-qPCR reaction. Each reaction system consisted of 10 μL SYBR qPCR Master Mix, 0.4 μL of forward primer, 0.4 μL of reverse primer, 1 μg of cDNA, and, finally, the volume was supplemented to 20 μL using ddH_2_O. The gene expression was analyzed using the ABI 7500 Real-Time PCR instrument (Applied Biosystems, Waltham, MA, USA), the RT-qPCR reaction process includes: 1. holding stage: 95 °C, 30 s; 2. cycling stage: each cycle contains 95 °C, 10 s; 60 °C, 30 s, total 40 cycles; 3. melt curve stage: 95 °C, 15 s; 60 °C, 60 s; 95 °C, 15 s. The quantitative reaction was carried out using Vazyme ChamQ Universal SYBR qPCR Master Mix (Vazyme, Nanjing, China). The primers used for the reaction were displayed in Table S1, and TaActin (wheat *β-Actin* gene [24]) was used as the internal reference gene [25]. The relative expression level of the target genes was calculated using the ΔΔCT method; the technique was repeated 3 times per sample.

### 2.8. Enzyme Activity Test

The samples were derived from leaf and root tissues in 2.7 and stored at −80 °C. The materials were fully ground using a mortar pre-cooled with liquid N, collected in a 2 mL EP tube, and NADH-GOGAT extract buffer was added (0.1 M phosphate buffer, pH = 7.5). The homogenate was centrifuged at 4 °C and 8000× *g* for 10 min. The supernatant was collected and the enzyme activity was determined using the NADH-GOGAT kit (GOGAT-2-Y) at 340 nm [26]. The Eppendorf Centrifuge-5810R and BECKMAN COULTER DU-640 nucleic acid and protein analyzer were utilized to handle and measure the absorbance values of the samples. The technique was repeated 3 times for each assay and were incorporated into the enzyme activity detection kit purchased from Comin Biotechnology Co. Ltd., Suzhou, China (http://www.cominbio.com/index.html (accessed on 10 May 2024)).

## 3. Results

### 3.1. Identification of TaGOGATs and Analysis of Gene Family Information

According to the Pfam code of the conserved domain (PF01645), two methods, HMM and BLAST, were used to query the members of *TaGOGATs* gene family. Six wheat glutamate synthase gene sequences were identified after screening. According to the chromosome positions and the type of electron donor, three of the genes annotated as *Fd-GOGAT* were named *TaGOGAT2s* (*TaGOGAT2-A*, *TaGOGAT2-B*, *TaGOGAT2-D*), and the other three *NADH-GOGAT* were named *TaGOGAT3s* (*TaGOGAT3-A*, *TaGOGAT3-B*, *TaGOGAT3-D*).

The physicochemical characteristics of TaGOGATs were analyzed. TaGOGATs with the same electron donor had similar characteristics. The molecular weight of the TaGOGAT2s was about 170,000 D, which was smaller than the 230,000 D of TaGOGAT3s. However, the molecular weight was reversed with the exon number, as TaGOGAT2s with a low molecular weight had more exons at 33 to 34 (Table 1). The instability coefficient analysis showed suggested that TaGOGAT3s had higher protein stability, while TaGOGAT2s was an unstable protein. Subcellular localization prediction showed that except for TaGOGAT3-D located in the mitochondria, the other five TaGOGATs were all located in chloroplasts. The results of the six TaGOGATs in terms of the protein isoelectric point, protein hydrophobicity, and transmembrane structure prediction manifested few differences; the isoelectric point ranged from 6.22 to 6.36, and all were hydrophilic proteins without a transmembrane domain (Table S2). These results indicate that TaGOGATs may have various biological functions.

### 3.2. Chromosome Localization Analysis of TaGOGATs

**Figure 1:**
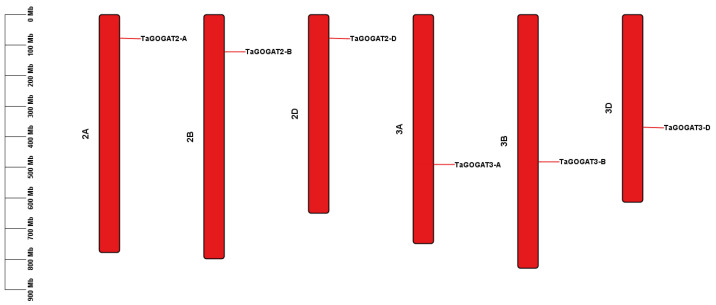
Location information of *TaGOGATs* on chromosomes. The six rectangles are the chromosomes. The chromosome names are labeled in the left side of the chromosome, and the gene name are marked on the right side. Chromosome-length information is provided in the scale at the far left of the image.

Common wheat was diploid (AA) in its ancestry, and after two natural hybridization and chromosome-doubling events in its evolutionary history, hexaploid wheat (AABBDD) was formed [27]. It contains 3 genomes (ABD), with a total of 21 chromosomes (Figure 1). Chromosome analysis showed that the six genes were located on the A, B and D genomes of chromosomes 2 and 3, respectively, and the relative positions of *GOGAT* genes with the same electron donor type were similar on chromosomes Chr 2A (*TaGOGAT2-A*), Chr 2B (*TaGOGAT2-B*), Chr 2D (*TaGOGAT2-D*), Chr 3A (*TaGOGAT3-A*), Chr 3B (*TaGOGAT3-B*) and Chr 3D (*TaGOGAT3-D*). The *TaGOGATs* gene family did not detect tandem and proximal duplication gene replication events.

### 3.3. Phylogenetic Tree Construction and Protein Conserved Motif Analysis of GOGAT

**Figure 2:**
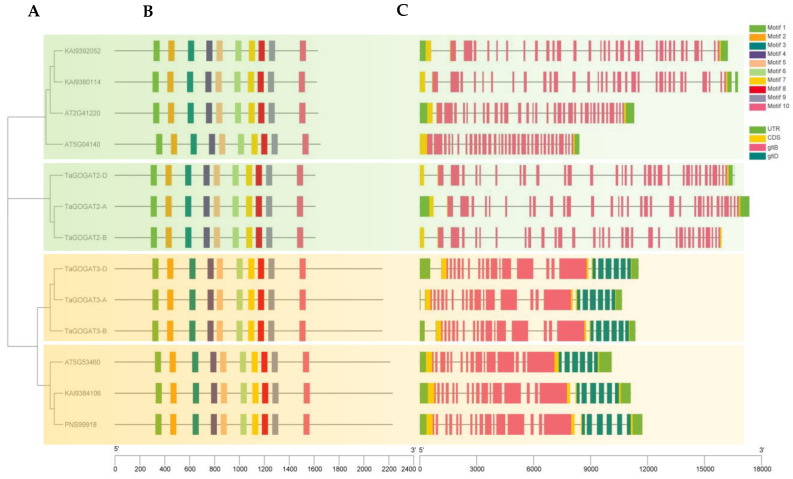

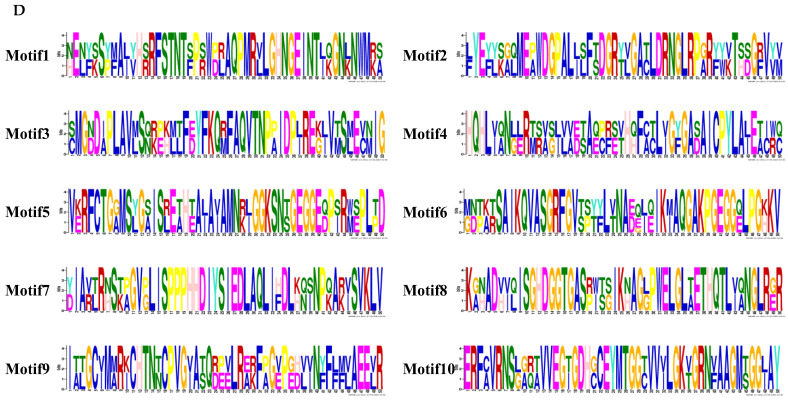
Phylogenetic tree, conserved motif, and gene structure analysis: (**A**) phylogenetic tree analysis. *Arabidopsis* (AT5G53460, AT5G04140, and AT2G41220), *Populus* (KAI9392052, KAI9380114, KAI9384106, PNS99918); (**B**) protein conserved motif map; (**C**) visualization of gene structure; and (**D**) conserved motif sequence.

The phylogenetic tree of the wheat and the reported GOGATs (Fd-GOGATs and NADH-GOGATs) from *Arabidopsis* (*Arabidopsis thaliana*) and *Populus* (*Populus trichocarpa*) was constructed for the analysis of the phylogenetic relationships. The results showed that TaGOGAT3s were clustered in a clade with the NADH-GOGATs from the other two species. TaGOGAT2s were clustered to a separate branch, indicating that the interspecies relationship of Fd-GOGATs in wheat is far greater than that of NADH-GOGATs. Moreover, TaGOGAT2-A and TaGOGAT2-B, TaGOGAT3-A and TaGOGAT3-D were more closely related (Figure 2A). Amino acid conserved motifs analysis showed that each GOGAT had 10 complete conserved motifs in the same order (Figure 2B). Visual analysis of the gene structure showed that NADH-GOGATs had two domains, gltB and gltD, while Fd-GOGATs had only the gltB domain. *TaGOGAT2-A* and *TaGOGAT2-B* had 33 exons, and *TaGOGAT2-D* had 34 exons. *TaGOGAT3-A*, *TaGOGAT3-B* and *TaGOGAT3-D* only contained 23 exons (Figure 2C). Six GOGATs of wheat had complete conserved domains (Figure 2B,C).

### 3.4. Collinearity Analysis of TaGOGATs Genes

**Figure 3:**
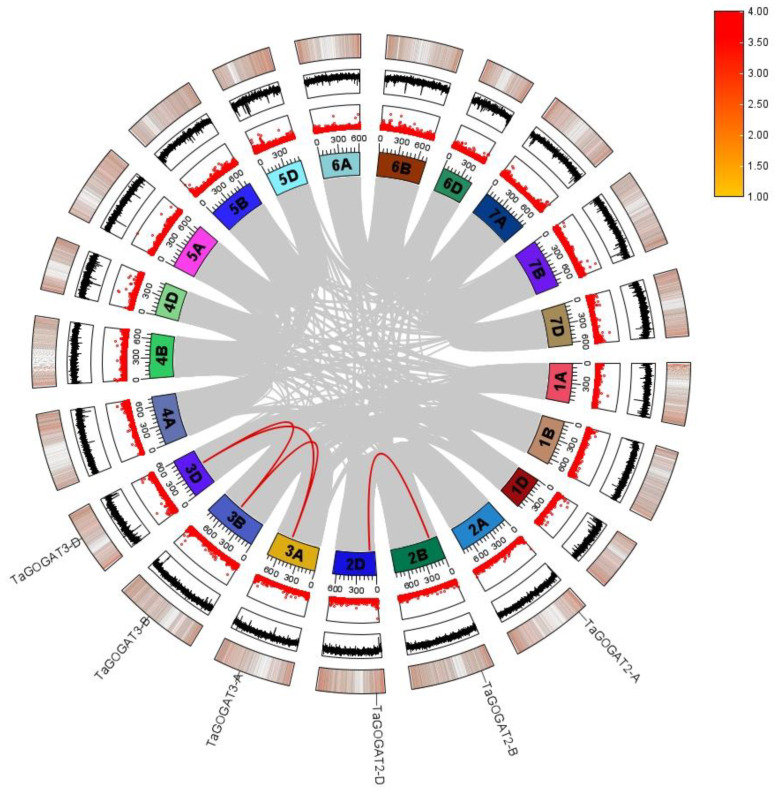
Collinearity analysis of *TaGOGATs* gene. The red lines indicate collinear gene pairs. The graph shows the gene name, gene density information, GC skew information, gene gap distribution information and chromosome name from the outside to the inside. The legend on the right corresponds to the size of the gene densities.

The Tbtools-II tool and MCScanX algorithm were used to explore the repetitive events of six *TaGOGATs* genes. The results showed that there are four collinear events in *TaGOGATs*, which are present on chromosomes 2B, 2D, 3A, 3B and 3D. *TaGOGAT2-A* did not have gene collinearity, while *TaGOGAT3-A*, *TaGOGAT3-B* and *TaGOGAT3-D* had gene collinearity events with each other (Figure 3). Repetitive events could be classified into 5 types according to gene replication types, which were tandem repeats, proximal repeats, transposed repeats, dispersed repeats, and single copies [28]. Both *TaGOGAT2-B/D* and *TaGOGAT3s* are transposed repeats, while *TaGOGAT2-A* is a dispersed repeat.

### 3.5. Ka/Ks Analysis of TaGOGATs

**Figure 4:**
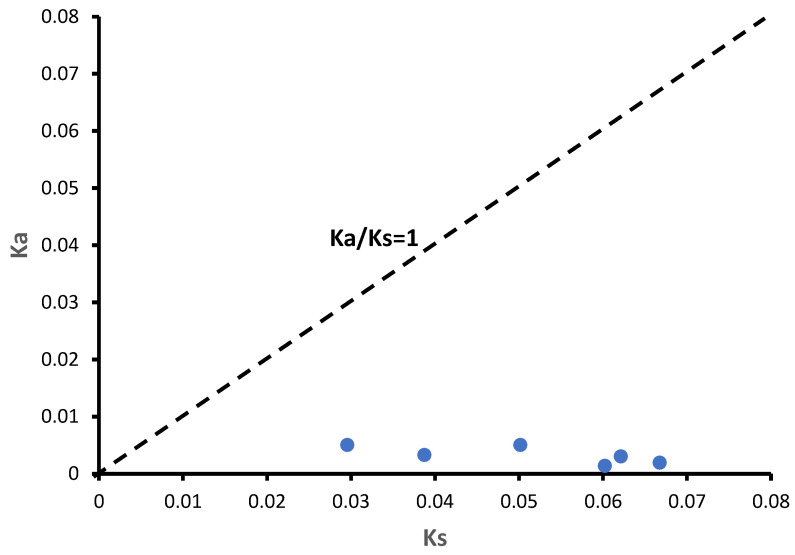
Ka/Ks scatter plot of *TaGOGATs* gene. Each point represents the coordinate of a gene pair.

Six *TaGOGAT* genes were classified into two branches by phylogenetic tree constructing. In order to study the *TaGOGATs* gene evolution was affected by selection pressure or not, the ratio of non-synonymous replacement rate (Ka) to synonymous replacement rate (Ks) was calculated. The results showed that the six genes had significant differences in Ks values, ranging from 0.029552 to 0.066745 (Table 2). The Ka/Ks ratios were all much less than 1, indicating that the related genes undergo the purifying selection. In most cases, selection eliminated harmful mutations and kept the protein sequence stable (Figure 4).

### 3.6. Analysis of Cis-Acting Elements of TaGOGATs Gene Promoter

**Figure 5:**
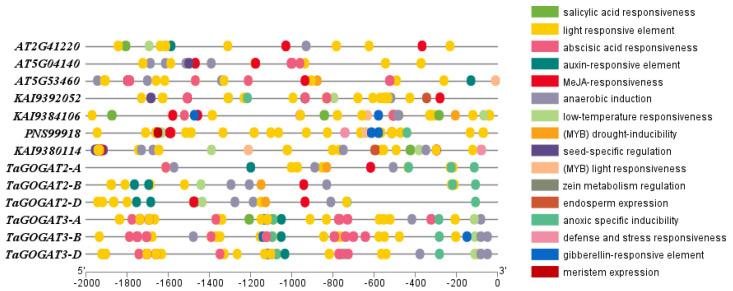
Prediction of promoter *cis*-acting elements. The 2 kb upstream region from the GOGATs translation start site in wheat, *Arabidopsis* and *Populus* were analyzed.

The *cis*-acting element is a DNA sequence on the promoter of genes that influences the gene expression. The 2kb upstream region from the *GOGATs* translation start site in wheat, *Arabidopsis* and *Populus* was analyzed for promoter *cis*-acting elements. The results showed that there was no obvious difference among species, but there was a significant discrepancy between *TaGOGAT2-A* and *TaGOGAT2-B/D*. The distribution regularity of *cis*-acting elements among *TaGOGAT3-A*, *TaGOGAT3-B* and *TaGOGAT3-D* is conservative, and the number of *cis*-acting elements in *TaGOGAT3s* is higher than that in *TaGOGAT2s* (Figure 5).

**Figure 6:**
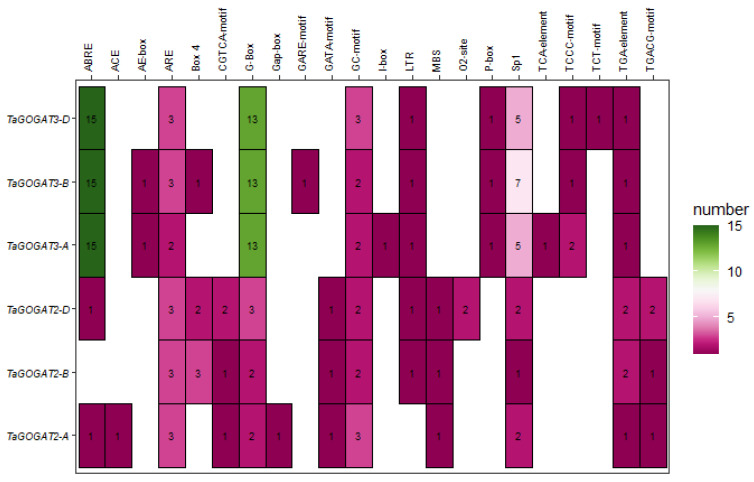
Predictive analysis of promoter *cis*-acting elements. In the figure, the vertical coordinate indicates the gene name, and the horizontal coordinate indicates the binding element name.

Many *cis*-acting elements related to hormone response, stress response, and developmental response processes were found in the promoter of *GOGAT* genes. Hormone response elements include abscisic acid response element (ABRE), auxin response element (TGA-element), gibberellin response element (P-box/GARE-motif), jasmonic acid response element (TGACG-motif/CGTCA-motif) and salicylic acid response element (TCA-element). Stress response related elements containing hypoxia-induced response elements (ARE/GC-motif), low-temperature response elements (LTR), drought-induced response elements (MYB binding site, MBS), light response dependent elements (AE-box/ACE/Gap-box/TCTmotif/TCCC-motif/GATA-motif/I-box/Sp1/Box4/G-Box). The *TaGOGATs* promoter region also contains several zein metabolic regulatory elements (O2-site), which are involved in plant biosynthesis and developmental related biological processes. The number of ABRE, G-box and sp1 in *TaGOGAT3s* promoters is obviously higher than that of other binding components, while the number of ARE binding components is higher in *TaGOGAT2* promoters compared with other promoters (Figure 6). In conclusion, the prediction results of *TaGOGATs cis*-acting elements indicate that *TaGOGAT* may be involved in biological metabolic processes such as growth and development, hormone response, and the stress response of wheat.

### 3.7. Multiple Amino Acid Sequences Analysis of TaGOGATs

The conserved sequences and functional regions of six TaGOGATs in wheat were analyzed. The results show that the six GOGAT proteins had high homology (Figure S1). The conserved binding regions have been reported in *Populus* GOGAT proteins [22], andcould also be found in TaGOGATs. A putative FMN-binding region (Figure S1B) and a putative [3Fe-4S] cluster-binding region were included in all proteins (Figure S1C). The putative NAD(P)H-binding regions was only contained in three NADH-GOGATs (Figure S1D).

### 3.8. Secondary and Tertiary Structure Characteristics Prediction of TaGOGAT Proteins

**Figure 7:**
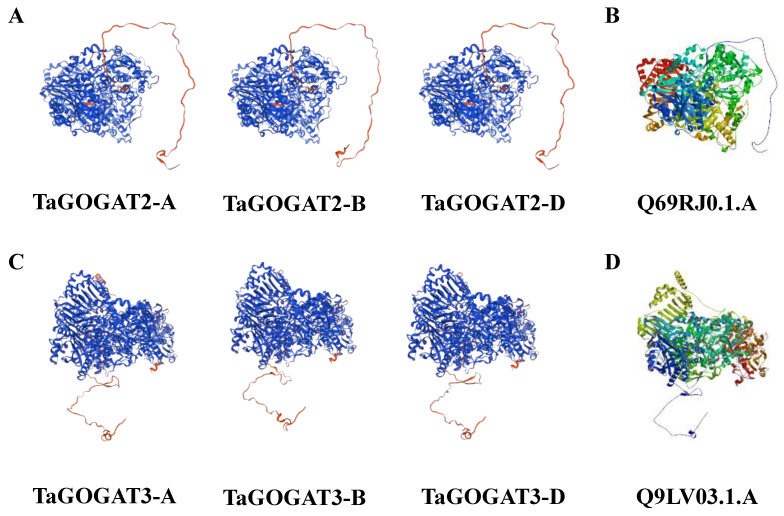
Tertiary structure prediction map of wheat TaGOGAT protein. Based on the homology model-building method, the optimal spatial structure model of the homologous proteins was predicted: (**A**) TaGOGAT2s protein tertiary structure diagram; (**B**) TaGOGAT2s protein optimum homologous protein template; (**C**) TaGOGAT3s protein tertiary structure diagram; and (**D**) TaGOGAT3s protein optimum homologous protein template. In (**A**,**C**), the blue peptide indicates that the amino acid consistency is greater than 70%, the red peptide indicates that the amino acid consistency is less than 50%, and the yellow peptide indicates that the amino acid consistency is 60–70%.

The tertiary structure model of TaGOGAT proteins was predicted by AFDB search and the AlphaFold v2 algorithm. The results showed that the optimal TaGOGAT2s template was Q69RJ0.1.A (Ferredoxin-dependent glutamate synthase). A Seq identity score greater than 90 indicated that the model had high homology with the comparison protein, and a global model quality estimate score (GMQE) greater than 0.9 indicated high data reliability (Figure 7A,B). Q9LV03.1.A (Glutamate synthase 1 [NADH]) was the optimal TaGOGAT3s template, with a Seq identity greater than 78 and a GMQE greater than 0.8 (Figure 7C,D). The PDB file of the optimal homology model for protein secondary structure analysis of TaGOGAT was downloaded on the ESPript 3.0 website. The results showed that the secondary structure characteristics of TaGOGAT2s were similar. This situation also existed in TaGOGAT3s (Table S3), which further verified the rationality of protein tertiary structure analysis at the level of the secondary structure.

### 3.9. Expression Patterns Analysis of TaGOGATs in Different Tissues

**Figure 8:**
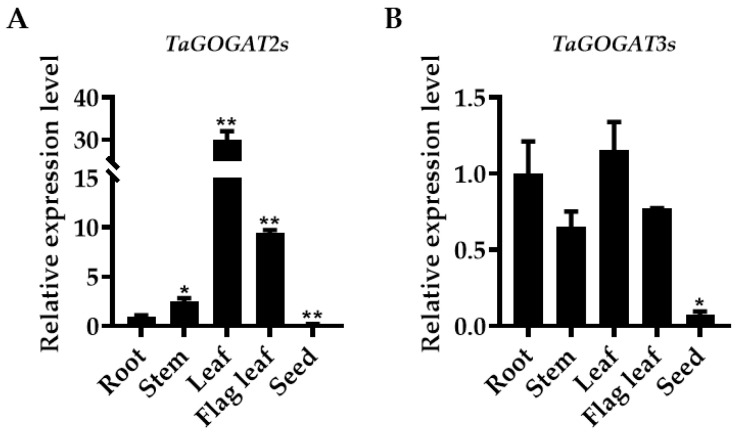
Analysis of *GOGAT* gene expression patterns in different tissues of wheat. RNA was extracted from wheat roots, stems, leaves, flag leaves, and seeds at the filling stage for RT-qPCR: (**A**) *TaGOGAT2s*; and (**B**) *TaGOGAT3s*. Sample significance was obtained based the on t-test method (*, 0.01 ≤ *p* < 0.05; **, *p* < 0.01).

To study the expression patterns of *TaGOGATs* in different tissues of wheat, RNA was extracted from the roots, stems, leaves, flag leaves and seeds of wheat at the filling stage, and the *TaGOGATs* expression level was analyzed by RT-qPCR. As *TaGOGATs* gene sequences located on the same chromosome are highly similar, two pairs of primers were designed to detect the gene expression levels of *Fd-GOGATs* located on chromosome 2 and *NADH-GOGATs* located on chromosome 3, respectively (Table S1). The results showed that *TaGOGATs* were expressed in different tissues of wheat. The *TaGOGAT2s* gene was mainly expressed in leaves and flag leaves, and was the lowest in seeds (Figure 8A). The *TaGOGAT3s* gene had the lowest gene expression level in seeds. The relative expression level of *TaGOGAT3s* in leaves was slightly higher than that in the root tissues, but it was not significant (Figure 8B). The results were similar to that in other species [22].

### 3.10. The Expression Analysis of TaGOGATs in Different Tissues of Wheat Seedling under N Deficiency Treatment

**Figure 9:**
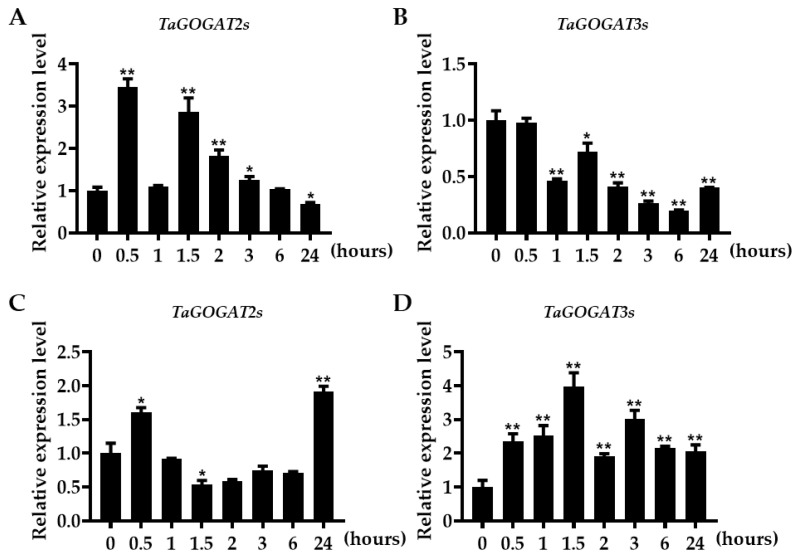
The relative expression analysis of *TaGOGAT* genes: (**A**) the relative expression analysis of *TaGOGAT2s* in wheat root tissue; (**B**) the relative expression analysis of *TaGOGAT3s* in root tissue; (**C**) the relative expression analysis of *TaGOGAT2s* in leaf tissue; and (**D**) the relative expression analysis of *TaGOGAT3s* in leaf tissue. Sample significance was obtained based on *t*-test method (*, 0.01 ≤ *p* < 0.05; **, *p* < 0.01).

TaGOGAT is one of the key enzymes in the wheat N metabolism pathway. In this study, RT-qPCR technology was used to analyze the changes in *TaGOGATs* gene expression levels in the root and leaf tissues of wheat seedlings under different N deficiency treatment times. The results showed that the expression of *TaGOGATs* gene was affected by the N hungry condition. In wheat root tissues, the expression level of *TaGOGAT2s* was upregulated by N deficiency induction, and induced to the highest level at 0.5 h, then gradually decreased to the pre-treatment level (0 h) at 6 h (Figure 9A). The *TaGOGAT3s* gene expressions were downregulated, but there were no significant changes at 0.5 h (Figure 9B). In leaf tissues, the relative expression level of *TaGOGAT2s* slightly increased at 0.5 h, then decreased, and suddenly reached a higher level at 24 h (Figure 9C). For *TaGOGAT3s*, the relative expression level gradually increased to the highest level at 1.5 h, and then decreased little by little, but was still higher than that in the pre-treatment level (Figure 9D). Overall, *TaGOGATs* could participate in the regulatory response of wheat N stress.

### 3.11. Analysis of TaGOGATs Enzyme Activity in Wheat Seedling Stage

**Figure 10:**
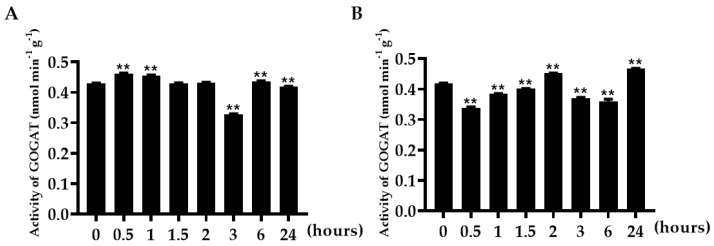
Activity analysis of TaGOGAT3s enzyme: (**A**) analysis of TaGOGAT3s enzyme activity in the root tissue of wheat seedlings; and (**B**) analysis of TaGOGAT3s enzyme activity in leaf tissue. Sample significance was obtained based on *t*-test method (**, *p* < 0.01).

The tissue-specific analysis of *TaGOGATs* showed that *TaGOGAT3s* can be expressed in different wheat tissue. The enzyme activity analysis showed that the enzyme activity in wheat root tissues was less affected by N stress, and suddenly decreased at 3 h, and then quickly recovered (Figure 10A). In leaf tissues, the change trend of TaGOGAT3s’ enzyme activity declined at 0.5 h, followed by a slightly increase with the increase in treatment, and then decreased at 3 and 6 h, and finally increasing to a high level at 24 h (Figure 10B).

## 4. Discussion

Currently, the *GOGAT* gene family were identified [22,29] in *Populus* and *Arabidopsis* species. Two *Fd-GOGAT* (*AT5G04140*, *AT2G41220*) and one *NADH-GOGAT* (*AT5G53460*) were identified in *Arabidopsis*. The poplar *GOGAT* gene family contains two *Fd-GOGAT* (*KAI9392052*, *KAI9380114*) and two *NADH-GOGAT* (*KAI9384106*, *PNS99918*). In wheat, six *GOGAT* genes were distinguished, which were located on chromosome 2 and chromosome 3, respectively. (Figure 2A). Through gene structure analysis, it was found that GOGAT of the same electron donor type had a similar domain distribution pattern. TaGOGAT2s contains only one gltB-conserved domain, while TaGOGAT3s contains gltB- and gltD-conserved domains, indicating that the two types of GOGAT may have biological functional diversity due to their different protein spatial structures (Figure 2C). Similar structures were found in *Arabidopsis* and *Populus* GOGAT genes. The *gltB* gene encodes the α subunit of GOGAT, and *gltD* is involved in encoding the β subunit [30]. Studies have shown that gltB and gltD are very important for maintaining GOGAT protein biological activity. *gltB* mutants of *Escherichia coli* are unable to utilize substances, such as proline and glycine, as the sole N source [31], and mutations of this gene in pea bacteroid RU2307 lead to an increase in the intracellular Gln: Glu ratio. Two amino acid uptake systems (Aap and Bra), both ABC-types, were inhibited [32]. The *gltD* gene mutants also showed similar characteristics. The *gltD::Tn5* mutant of *Bradyrhizobium* ORS285 could not use ammonium, nitrate, and many amino acids as N sources for its growth, and could not undergo the N fixation reaction under natural conditions [33].

These results suggest that the function of GOGAT is relatively conservative among species. By evolutionary tree analysis of 13 species, including prokaryotes and eukaryotes, Cao et al. found that only prokaryotes contain Fd-GOGAT, and then speculated that, in the evolutionary history of GOGAT, Fd-GOGAT appeared first, and the emergence of NADH-GOGAT resulted in functional differentiation [22]. Four *GOGATs* have been identified in *Populus*, and subcellular localization predictions show that they are all located in chloroplasts. Two of these proteins depend on Fd as electron donors, and the other two proteins have NADH. Interestingly, subcellular localization prediction analysis showed that TaGOGAT3-D were located in mitochondria, and the remaining five TaGOGAT proteins were all in chloroplasts, which may indicate that they were involved in different biological reactions. The Ka/Ks ratio between wheat gene pairs was found to be much less than 1 through gene analysis, indicating that *TaGOGATs* were subjected to purification selection in the evolutionary process, which also indicated that TaGOGATs were highly conserved, consistent with the previous conclusions (Figure 4). Analysis of *cis*-acting elements of gene promoters showed that *TaGOGATs* contained many different types of acting elements (Figure 5). For example, *TaGOGAT3s* contained several gibberellin-responsive elements (P-box, GARE-motif), indicating that it may be involved in gibberellin response regulating (Figure 6). In order to analyze the effects of abscisic acid (ABA), gibberellanic acid (GA3) and other hormones on the expression levels of two *Fd-GOGAT* (*GLU1* and *GLU2*) and one *NADH-GOGAT*(*GLT1*) in *Arabidopsis*, *Arabidopsis* seedlings treated with different hormone concentrations were analyzed. The results showed that, except for *GLU2*, GA3 enhanced the expression of other genes in shoots [34]. This is consistent with our analysis. Through amino acid sequence comparison analysis, it was found that the amino acid sequence of TaGOGAT with the same electron donor type was highly consistent (Figure S1); the spatial structure analysis of the proteins also led to the same conclusion (Figure 7). The FMN-binding sites and [3Fe-4S] cluster-binding regions were contained in all six TaGOGAT proteins. TaGOGAT3s included other predicted NAD(P)H binding sites. These binding sites have also been reported in *Populus* GOGAT-related studies [22], and the difference in the protein domain reflects the difference of its biological reaction process.

The GS/GOGAT cycle participates in plant N assimilation. Six *GOGAT* genes were isolated in bread wheat by the partial barley sequence [35] and *NADH-GOGAT* rice genome sequence [36], which were located on wheat chromosomes 2 and 3, respectively. The expression levels of *NADH-GOGAT*, except for *NADH-GOGAT-3D*, in durum wheat were correlated with grain protein content [37]. *NADH-GOGAT*, located on wheat chromosomes 3A and 3B, were identified as a major candidate for NUE by meta-QTL. NO_3_^−^ and NH_4_^+^ are the main forms of inorganic N absorbed by plants, and their ion concentration and morphology could influence the expression of plant *GOGAT* genes. *NADH-GOGAT* (*OsGlt1* and *OsGlt2*) were mainly expressed in rice root tissues under N-restricted treatment, but in leaves under unrestricted conditions [38]. The expression and activity of GOGAT and GS could be regulated by the ratio of ammonium–nitrogen, and the relationship between them showed a significant positive correlation [39]. Both high and low concentrations of NH_4_^+^ could inhibit the expression of *GOGAT* gene. High NH_4_^+^ concentrations could produce toxicity and inhibit the N assimilation process [40]. Many studies have shown that *Fd-GOGAT* is mainly expressed in photosynthetic tissues, while *NADH-GOGAT* is expressed in non-photosynthetic plastid tissues [41]. The transcription levels of six *GOGAT* genes in wheat roots, stems, leaves, flag leaves and seeds were tested; it was shown that *TaGOGAT2s* were mainly expressed in leaves, while *TaGOGAT3s* were highly expressed in roots (Figure 8). *GOGAT* gene expression in wheat seedlings had been detected under N deficiency treatment. It was found that the expression level of *TaGOGAT2s* in root tissues increased first, induced by N deficiency, and then decreased, and that it was the highest at 0.5 h and decreased to the pre-treatment level at 24 h (Figure 9A). This pattern can also be observed in the regulation of *TaGOGAT3s* gene expression in leaf tissues, with the highest transcription level at 1.5 h followed by a gradual decrease thereafter (Figure 9C). 

However, the transcription levels of *TaGOGAT3s* in wheat root tissues showed an overall downward trend (Figure 9B). It was reported that plant hormone abscisic acid (ABA) could enhance the bioactivity of GS and GOGAT, thereby reducing NH_4_^+^ accumulation and cellular oxidative damage [42]. TabZIP60 could bind to the ABRE elements on the *TaNADH-GOGAT-3B* promoter and negatively regulate its expression [43]. ABA-responsive *cis*-acting elements (ABRE) were found in all gene promoters except *TaGOGAT2-B* (Figure 5 and Figure 6). However, *GOGAT* was the highly tissue-specific gene. Thus, the down-regulated expression of *TaGOGAT3s* in root tissues under N deficiency conditions may be related to the recovery of NH_4_^+^ concentrations in plants. Enzyme activity in TaGOGAT2s is highly correlated with light source and external sucrose induction [44]. The effect of N starvation induction on enzyme activity in TaGOGAT3s in wheat leaf and root tissues were studied. Enzyme activity analysis showed that the enzyme activity in TaGOGAT3s in wheat leaf tissue was significantly negatively regulated (Figure 10B). However, in wheat root tissues, the change in enzyme activity level was less affected by N stress (Figure 10A). Balotf et al. found that gene expression and enzyme activity in GOGAT were inhibited in wheat leaf tissue after 7 days of cultivation under nitrogen-starved conditions [45].

## 5. Conclusions

As one of the basic elements of an organism, N is very important for the growth and metabolism of wheat. GOGAT, as a key enzyme in the process of plant N assimilation, affects wheat N absorption efficiency. In this study, we identified the *TaGOGATs* gene family through bioinformatics analysis and identified a total of six genes, which were located on chromosomes 2 and 3. *TaGOGAT2s* was mainly expressed in leaves and flag leaves, while *TaGOGAT3s* was mainly expressed in roots and leaves. the expression of the *GOGATs* gene could be induced in leaves and roots under the treatment of N deficiency. Enzyme activity in TaGOGAT3s in wheat leaf tissues was significantly affected by N deficiency stress, and the regulation mechanism of enzyme activity in leaf and root tissue may be different. This paper provides a systematic approach for *TaGOGAT* gene family analysis. The research results indicate that *TaGOGAT* may participate in regulating wheat NUE.

## Figures and Tables

**Table 1:** The basic nucleic acid and protein information of *TaGOGAT* genes in wheat.

Gene Name	Locus Name	Chromosome Location	Exons Number	Gene Length (bp)	Amino Acid Length (aa)	Molecular Weight (Da)
*TaGOGAT2-A*	TraesCS2A02G130600	Chr 2A: 78328734-78346097	33	5805	1606	174,146.2
*TaGOGAT2-B*	TraesCS2B02G152900	Chr 2B: 121354806-121371191	33	5309	1607	174,285.35
*TaGOGAT2-D*	TraesCS2D02G132900	Chr 2D: 78375985-78392563	34	5051	1606	174,193.26
*TaGOGAT3-A*	TraesCS3A02G266300	Chr 3A: 490922100-490932750	23	6854	2152	235,258.73
*TaGOGAT3-B*	TraesCS3B02G299800	Chr 3B: 481595302-481606660	23	7307	2145	234,364.58
*TaGOGAT3-D*	TraesCS3D02G266400	Chr 3D: 369790549-369802074	23	7383	2145	234,534.95

**Table 2:** Ka/Ks analysis of gene pairs.

Gene 1	Gene 2	Ka	Ks	Ka/Ks
*TaGOGAT3-A*	*TaGOGAT3-B*	0.005017	0.050165	0.100013
*TaGOGAT3-D*	*TaGOGAT3-A*	0.003273	0.038757	0.084443
*TaGOGAT3-D*	*TaGOGAT3-B*	0.005021	0.029552	0.169886
*TaGOGAT2-A*	*TaGOGAT2-B*	0.001924	0.066745	0.028821
*TaGOGAT2-D*	*TaGOGAT2-A*	0.001374	0.060223	0.022812
*TaGOGAT2-D*	*TaGOGAT2-B*	0.003025	0.06212	0.048695

## Data Availability

Data are contained within the article or the Supplementary Materials.

## Supplementary Materials

The following supporting information can be downloaded at: https://www.mdpi.com/article/10.3390/genes15070827/s1, Table S1: RT-PCR Primer sequence; Table S2: Information of wheat glutamate synthase family members; Table S3: The number of the secondary structure type in TaGOGAT proteins. Figure S1: Amino acid sequence alignment of wheat glutamate synthase TaGOGATs.
