# Supplementary material for: Identification of Wheat Glutamate Synthetase Gene Family and Expression Analysis under Nitrogen Stress
Source: Genes (Basel). 2024 Jun 22;15(7):827. doi: 10.3390/genes15070827 (PMC11275450; doi:10.3390/genes15070827)
Supplement: Supplementary file 1 [file genes-15-00827-s001.zip › genes-3051080-supplementary.pdf]

Table S1 RT-PCR Primer sequence

Gene name	Primer F	Primer R
TaActin	TGCTATCCTTCGTTTGGACCTT	AGCGGTTGTTGTGAGGGAGT
TaGOGAT2s	CGAGGCAACATCTTGGAGGT	CAGCAGCATCAGCTTCTTCAC
TaGOGAT3s	GCACCAGGAAGCATCTACCA	CAAGGAGGACAAGGTCTGCC

Table S2 Information of wheat glutamate synthase family members

Gene Name	Isoelectric Point	GRAVY	Instability Index:	Subcellular Location	Transmembrane Structure
TaGOGAT2-A	6.25	-0.153	40.57	Chloroplast	None
TaGOGAT2-B	6.25	-0.155	40.72	Chloroplast	None
TaGOGAT2-D	6.3	-0.151	40.94	Chloroplast	None
TaGOGAT3-A	6.29	-0.261	37.15	Chloroplast	None
TaGOGAT3-B	6.22	-0.267	36.96	Chloroplast	None
TaGOGAT3-D	6.36	-0.263	36.87	Mitochondria	None

Table S3 The number of the secondary structure type in TaGOGAT proteins

Type	TaGOGAT2-A	TaGOGAT2-B	TaGOGAT2-D	TaGOGAT3-A	TaGOGAT3-B	TaGOGAT3-D
310-helices	22	23	19	28	28	30
α-helices	50	50	50	73	73	72
β-strands	52	52	50	67	65	67
α-turns	2	5	3	1	22	2
β-turns	29	28	30	43	45	40

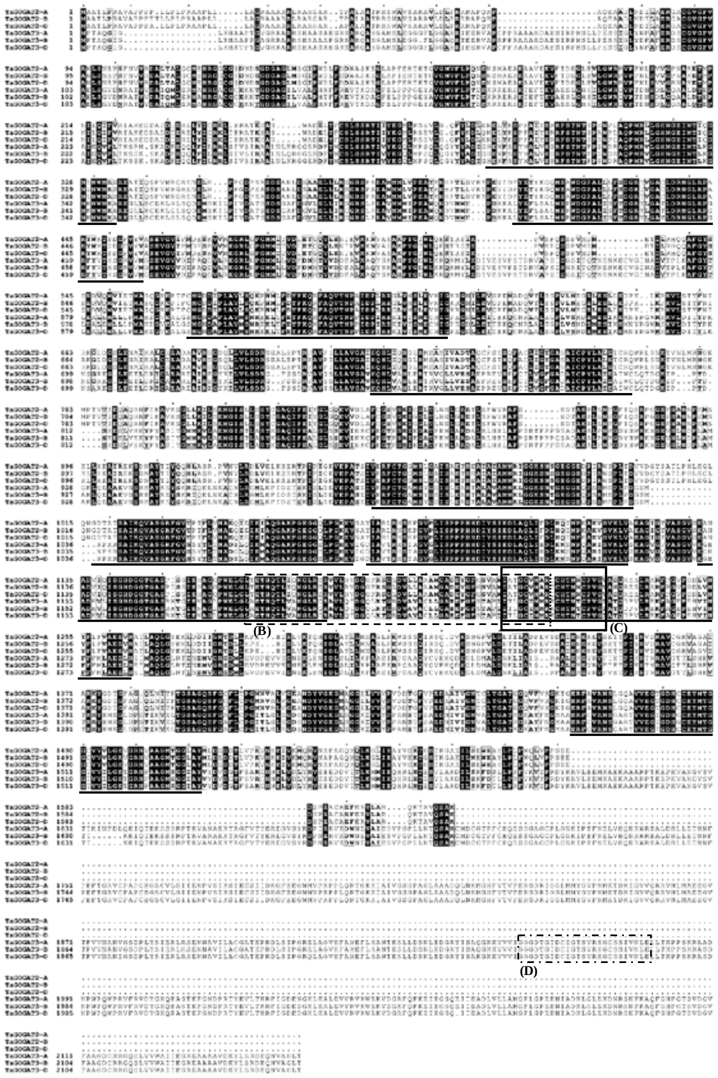
(B)
(C)
(D)

Figure S1. Amino acid sequence alignment of wheat glutamate synthase TaGOGATs. (A) Black lines are drawn at the bottom of the GOGAT conserved motifs sequence (B) The box is formed by the solid line, putative FMN binding region. (C) The box is formed by dashed line, putative [3Fe-4S] cluster binding region. (D) Boxes formed by dots and dashed lines, putative NAD(P)H binding region of NADH-GOGATs.
